# ArControl: An Arduino-Based Comprehensive Behavioral Platform with Real-Time Performance

**DOI:** 10.3389/fnbeh.2017.00244

**Published:** 2017-12-11

**Authors:** Xinfeng Chen, Haohong Li

**Affiliations:** Department of Biomedical Engineering, School of Life Science and Technology, Huazhong University of Science and Technology, Wuhan, China

**Keywords:** behavioral platform, Arduino, State notation, methods, software, Go/No-Go, real-time system

## Abstract

Studying animal behavior in the lab requires reliable delivering stimulations and monitoring responses. We constructed a comprehensive behavioral platform (ArControl: Arduino Control Platform) that was an affordable, easy-to-use, high-performance solution combined software and hardware components. The hardware component was consisted of an Arduino UNO board and a simple drive circuit. As for software, the ArControl provided a stand-alone and intuitive GUI (graphical user interface) application that did not require users to master scripts. The experiment data were automatically recorded with the built in DAQ (data acquisition) function. The ArControl also allowed the behavioral schedule to be entirely stored in and operated on the Arduino chip. This made the ArControl a genuine, real-time system with high temporal resolution (<1 ms). We tested the ArControl, based on strict performance measurements and two mice behavioral experiments. The results showed that the ArControl was an adaptive and reliable system suitable for behavioral research.

## Introduction

Animals have the ability to optimize their behavior when subjected to environmental change. Pavlovian conditioning and operant conditioning showed that animals adapt their behavior to the regular environmental stimulations. Researchers are able to assess the learning and the plasticity in psychology and functional neuroscience through this conditioning behavior. In order to implement these conditioning approaches, it is important to design a powerful platform that masters various reinforcement schedules, and precisely monitors animal's response and stimulation delivery.

The “Skinner box” (Skinner, 1938) enabled the development of many conditioning behavioral systems that design reinforcement schedules and record the data. However, the commercially available platforms are costly. Most of open-source systems are respectively restricted to a certain schedule. For example, there is a signal generator and recorder (D'Ausilio, 2012), a rodent visual discriminative task system (Pineño, 2014), an auditory discriminative task system (Ribeiro et al., 2017), a sucrose preference test system (Devarakonda et al., 2016; Longley et al., 2017), a nose poke system (Rizzi et al., 2016), and a Go/No-Go task system (Micallef et al., 2017). Many of these systems lack the GUI (D'Ausilio, 2012; Devarakonda et al., 2016; Rizzi et al., 2016; Longley et al., 2017; Micallef et al., 2017). In addition to the high costs and lack of flexibility, time accuracy is a major hurdle within the operant systems parameters. Low temporal accuracy results in loss of recording transient signals, which consequently induces inappropriate responses. Since the task-switch effects in the computer are unpredictable, it becomes an inherent defect in the common software that operates on a strict timing schedule, particularly when the host computer becomes over-loaded (Escobar and Pérez-Herrera, 2015).

To address these issues, we developed an inexpensive and powerful behavioral platform—the ArControl (Arduino Control Platform). The hardware was composed of a low-cost, yet high-performance Arduino chip, and a simple driver circuit. The software in the computer provided easy-to-use GUI applications, which allowed users to graphically program multipurpose tasks and subsequently acquire experimental data without mastering any textual scripts. The programmed task was both stored in and operated on the Arduino board. This allowed the ArControl to be a genuine real-time system, with high temporal accuracy, and free from the computer load.

Through both the technical parameters assessment and the practical behavioral experiments in mice, our ArControl platform was proven to be reliable and adaptive within various behavioral tasks. The source code and the PCB drafts are open-source (see https://github.com/chenxinfeng4/ArControl) under a creative commons license (GNU LGPL v2.1).

## System overview

**Figure 1:**
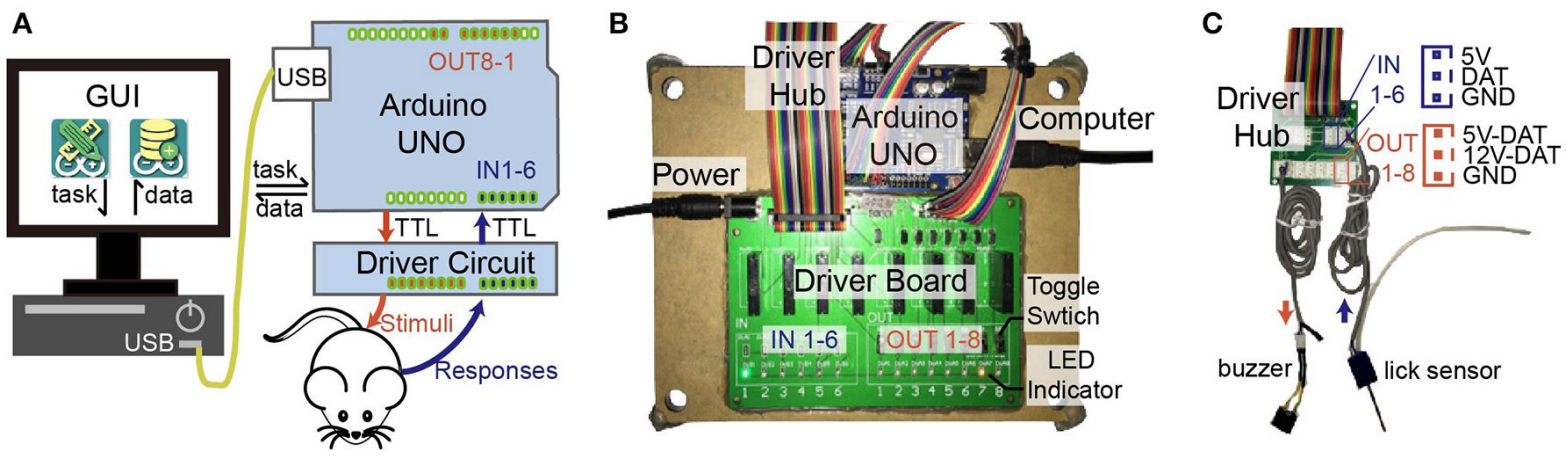
Diagram of ArControl. **(A)** Hierarchic structure of ArControl. Arduino is the core pivot managing devices, operating tasks, and logging data. The host computer has corresponding GUI assistance. **(B)** Hardware of ArControl consists of an Arduino Uno Rev3 and a driver board for voltage conversion. **(C)** Driver hub provides slots for terminal devices. Sensors can work at 5 V, and stimulators can work at 5 or 12 V.

The goal of the ArControl was to establish an Arduino-based behavioral platform that could control the devices to deliver stimulations and monitor behavioral responses (Figure 1A). The basic features of this platform were: (1) comprehensive—a combination of software and hardware, behavioral task design, and experimental data collection; (2) inexpensive—neither dedicated nor expensive hardware was required; (3) flexibility—the ArControl was applicable to multiple behavioral tasks; (4) easy to use—behavioral task could be designed using the State Notation principle through a user-friendly GUI, without the need to master the script language; (5) real-time performance—it had a high temporal resolution and was free from the computer load.

The ArControl platform centered around the Arduino board and the State Notation principle. For one part, the Arduino board was the main component that managed input and output devices, stored and executed the behavioral task, and recorded experimental data. The devices driving was allocated to the inferior driver circuit. The task design and the data collection was allocated to the superior PC (personal computer). For another, the State Notation principle, a derivation of the FSM (finite state machine), is a widely accepted in commercial platform, seen in MedState Notation (http://www.medassociates.com), Graphic State Notation (http://www.coulbourn.com), and LabState (http://anilab.bioon.com.cn). We applied this principle to the ArControl software, which allowed behavioral tasks to be constructed via intuitive graphical operations within the PC.

### Hardware structure

The hardware of ArControl consisted of the Arduino UNO Rev3 board and the driver circuit (Figure 1A). The Arduino UNO ports were simplified as the TTL-inputs and the TTL-outputs, which mastered the digital-switch signals. The input and output devices were marked as IN 1–6 and OUT 1–8, where they were identically managed through the Arduino and powered by the driver circuit. Multiple ArControl platforms could be connected to a single PC via USB cables.

Our driver circuit was divided into two parts. The driver board supplied the voltage conversion that enabled the terminal devices to work at 5 or 12 V (Figure 1B). The driver hub supported uniform slots for the input sensors and the output simulators connections (Figure 1C). This driver board provided LED indicators and toggle-switches to reflect and change the Arduino status. Users are encouraged to modify and reconstruct the driver circuit from our template.

The ArControl had been tested on regular Microsoft Windows PCs (windows 7/10, core-i5 CPU, 4 GB Memory). The ArControl platform costs around RMB ¥300 (US $45; excludes training chamber, sensors, and stimulators) to construct. The animal behavioral results in this paper were achieved solely using this platform.

### Software structure

**Figure 2:**
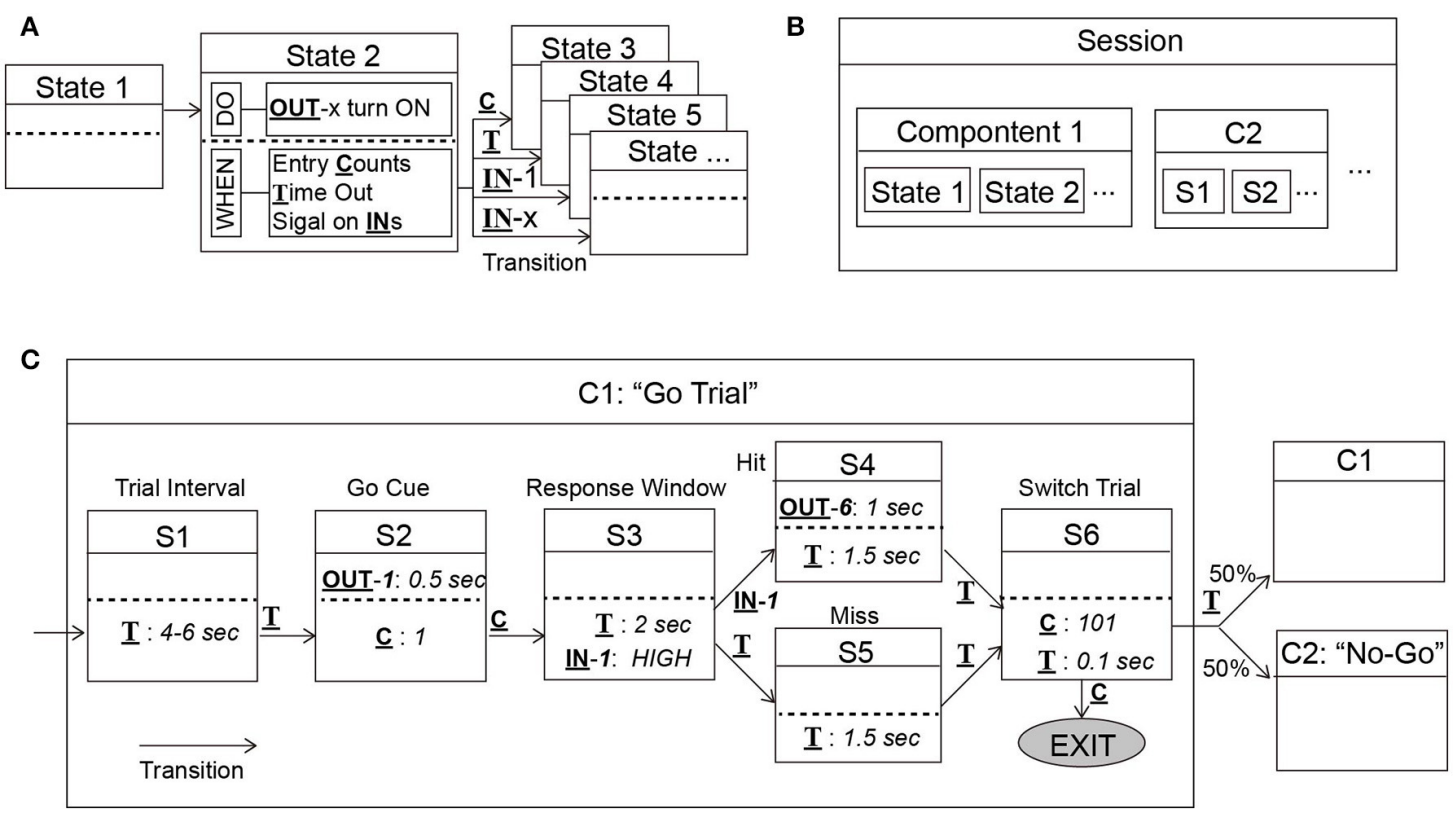
Diagram of the State Notation principle utilized by ArControl. **(A)** The State has a *do-function* to deliver stimulus, and *when-functions* that master the transitions cross States. (**B**) Component (abbr. C) and Session structures are the primary and the secondary collection of States (abbr. S). **(C)** Illustration of a Go Trial in the Go/No-Go task.

The highlight of ArControl was the use of the State Notation principle in order to construct sequential procedures of a task diagram. A behavioral task could be homogeneously decomposed as a sequence of States, where each State played a relevant role within the task. The State was a stand-alone object defining “do something for outputs,” “detect triggers,” and subsequently “respond to a trigger by switching to a corresponding next-State.” Namely, the State was a package of the *do-function*, the *when-function*, and the *transition-function* (Figure 2A). Through an identical State frame (Figures 2C, **5A**), this principle was capable to handle diverse behavioral procedures, such as presenting stimuli, detecting sensor signal, waiting until time-out, and counting trials. In general, the State Notation enabled a schedule to be programmed by assigning elements into box-frames, rather than by textual scripts. This was a convenient and powerful tool for constructing multipurpose behavioral tasks.

To implement the State Notation principle in a behavioral task design, the ArControl provided GUI assistance (ArControl Designer) for the State modeling. Subsequently, the States diagram of the behavioral task was automatically translated into the executable Arduino script file. The ArControl entirely stored the task and performed the task on the Arduino chip alone, and eventually became a real-time system. Lastly, the task was executed in and recorded from Arduino, with an additional GUI assistance (ArControl Recorder).

#### ArControl designer

**Figure 3:**
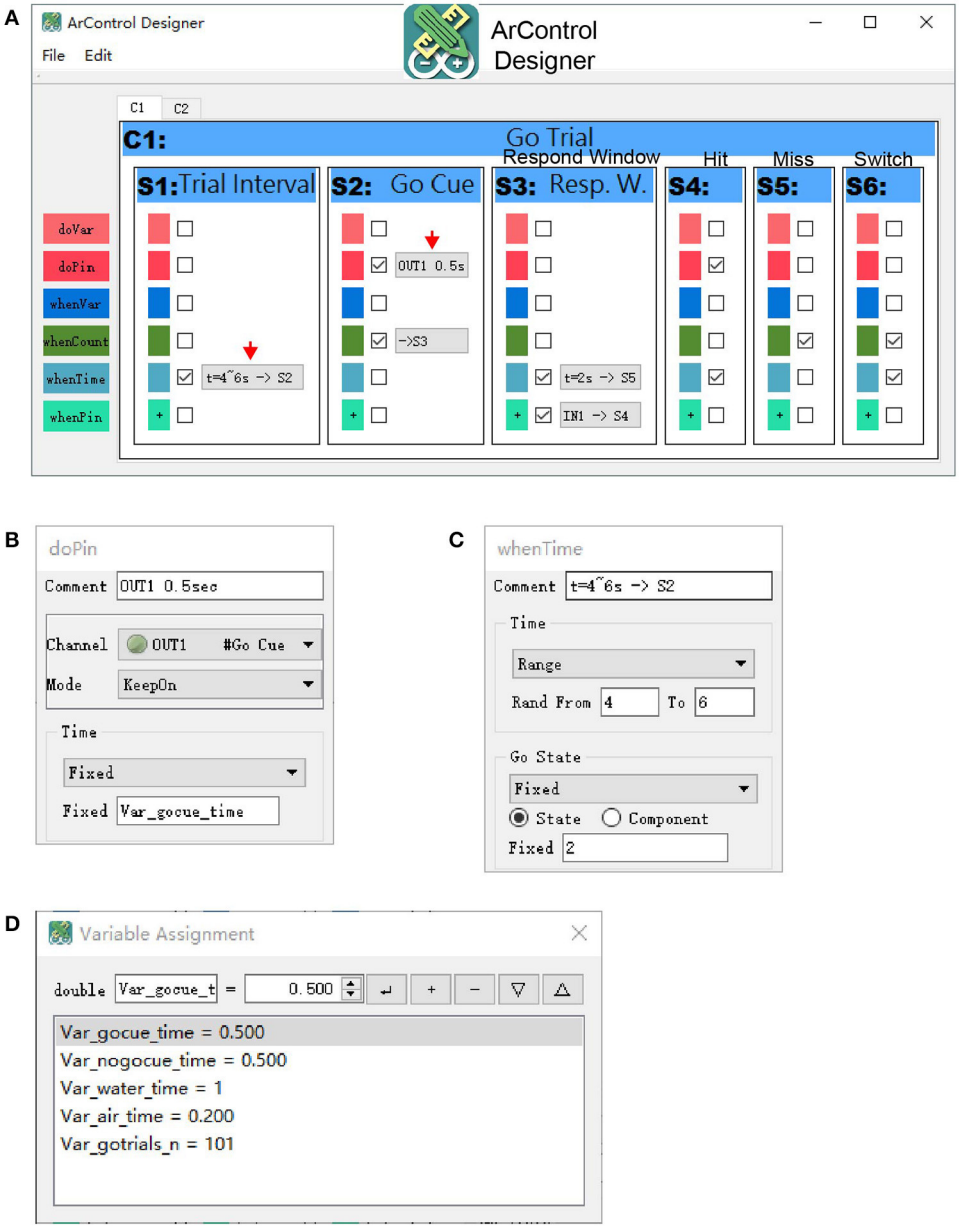
ArControl Designer for task design. **(A)** Main window of the ArControl Designer shows an implementation of the Go/No-Go task. **(B,C)** Typical pop-up windows to configure a *do-function* **(B)** and a *when-function* **(C). (D)** Window defines and initializes the Global Variable.

The ArControl Designer was the core application for the construction of the behavioral task. The main layout itself intuitively mimicked the diagram of the State Notation modeling (Figure 3A). Different States could be added and deleted in the GUI. Further details of a State were configured through several pop-up windows (Figures 3B,C).

##### State output

The State Output was also called the *do-function* (Figures 2A, 3A,B). Each State could proactively activate a specific output channel (OUT1-8) to present the animal with a stimulus (denoted as *do-pin*). There were two available basic output patterns—*turning on/off* for once and *keeping on* for a specified time.

##### State transition

The State Transition (Figures 2A, 3A,C) specified when the current State should be exited (*when-function*), and which corresponding State should be subsequently entered (*transition-function*). There were three types of transition conditions. The State Entry Transition (*when-count*) worked when the current State's entry had been counted for the specified number of times. The Input Channel Transition (*when-pin*) worked when the desired input channel (IN1-6) was activated. The Time Out Transition (*when-time*) defined the maximum survival time of the State if none of the previous conditions were activated.

There was only one State running at any moment. When the current State matched the conditions above, it made transition to the next State, next Component, or Session Exit.

##### Global variable

Users sometimes need to methodically manage important parameters for a schedule, such as trial counts and stimulation time. It was a good practice to apply the Global Variable (Figure 3D). Almost every parameter of the State frame supported the variables (Figure 3C). These variables were sharable among the States and could be modified dynamically during the task execution. Therefore, they could work as transmitters that allowed States to communicate between each other.

##### Advanced state output and transition

The Traditional State Output and the State Transition was rigid when defining a complex output pattern and a complicated transition condition. In this case, the ArControl retained the user-script interfaces for experts to master the custom functions. For general users, these features were simply used to manage the Global Variable mentioned above (thereby named *do-var* and *when-var)*.

##### Hierarchy of state, component, and session

A task could be arranged as a series of States, although it would be beneficial to group similar States into hierarchic structures. The Component was a virtual collection of States that benefited the States arrangement (Figure 2B). For example, the Go/No-Go task (**Figure 5A**) considered the Go Trial as a part of Componet-1, and the No-Go Trial as a part of Component-2 (Figures 2C, 3A). In the same way, the Components formed the Session, which was a technical term that represented the schedule and the task.

#### ArControl recorder

The basic functions of the ArControl Recorder was clicking to start/stop the running of a ready Session, as well as collecting and displaying the data flow (Supplementary Figures S1A–C). A useful tool (Firmata) was provided to directly debug the input and the output devices.

#### Data collection

The Arduino was limited in that the DAQ progress induced transient time-blocking. The ArControl had to compromise the executive ability with the completeness of the experimental data collection. This procedure was configured in the ArControl Designer, as the Record Level (Level 1–3). Level 1 had no data collection and achieved the quickest executive efficiency (temporal resolution <0.1 ms). Level 2 only collected the State information and obtained tiny blocking. Level 3 had comprehensive data collection and obtained the most blocking (temporal resolution <1 ms). Detailed measurements of the blocking within ArControl Levels are shown in the section below.

**Figure 4:**
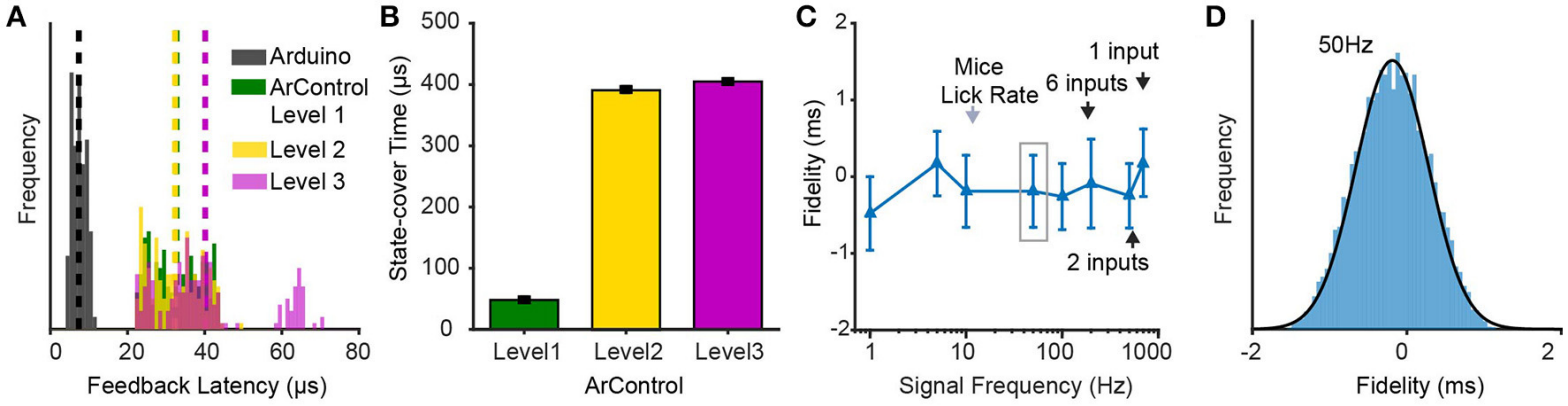
Performance verifications of ArControl. **(A)** Feedback latency from detecting an input to delivering an output. *Arduino* represents the native Arduino environment. ArControl *Level 1-3* are gradient tradeoffs between execution efficiency and data collection. **(B)** Time-consumption of an empty State. **(C)** DAQ ability in the ArControl *Level 3*. Signals beyond the frequency (black arrow) fail to be accurately reported. **(D)** Details of the left gray box in **(C)**. Data are mean ± *sd*.

Nonetheless, Level 3 was the most suitable for animal behavioral experiments. Animal behavior occurred on a macro-scale against milliseconds, resulting in the side effect imperceptible for behavioral researches. In Level 3, the data was comprehensively recorded in a way that all connected TTL-inputs were recorded, whether they were involved in controlling state flow or not. Additionally, every output event and state transition were acquired (Supplementary Figure S1B). The precision of data was specified as millisecond with the accuracy of ±1 ms (Figures 4C,D), which was acceptable for general usage.

Each running result of a session was saved in a subject-specific location, as a single ASCII format file (Supplementary Figure S1C). This data could easily be translated into a Matlab MAT-file (in our software package). This provided users with convenient offline analyses.

## Technical performance verification

In addition to cost and flexibility, behavioral platforms also require high performance that allows them to accurately master input responses and output stimuluses. Both the time accuracy and the DAQ ability were tested on the ArControl. The results demonstrated that the ArControl had the temporal resolution of <1 ms and could accurately record up to 200 Hz input signals. This technical performance of ArControl was adequate for behavioral research.

### Feedback latency

The feedback latency represents the time that it takes to detect an input signal and then to emit the feedback output signal. To measure this, a Session that consisted of two States was constructed. State-1 configured a transition to State-2 when detecting an input signal (e.g., IN1 for HIGH). State-2 then turned on an output (e.g., OUT1 for HIGH). The lag between the onset of IN1 and OUT1 denoted the feedback latency (Figure 4A).

The ArControl took 40 μs to accomplish the feedback, which was far quicker than regular counterparts, most of which obtain around 1 ms accuracy (Zhang, 2006; Escobar and Pérez-Herrera, 2015). The commercial platform LabState regularly took 20 ± 1 ms (±*sd*) to do this feedback stimulation. The diverse Record Levels acted similarly, although a bit more time was taken in Level 3, as a result of the DAQ module.

### State-cover time

The State Notation programming was powerful and flexible, however, the State structure itself was time-consuming. Even an empty State took a certain time, which was referred to as the State-cover time. We constructed a null State-1 with an immediate transition to State-2. The State-cover time was calculated via subtracting the start time between State-1 and State-2 (Figure 4B).

There was an observable difference between the ArControl Level 1 and Level 2/3. Consistent with previous announcement, the Level 1 was the most efficient. The time cost in Level 2/3 was higher, which resulted from the DAQ process. Generally, the ArControl took only 400 μs for the State-cover time, which met the common requirements.

### DAQ ability

The ArControl had an inbuilt DI/DO/State-Transition recorder module at Level 3, which could be considered as a light DAQ. Since the inputs and the outputs were identically represented as the TTL signals, this DAQ module achieved a high sampling rate.

The results proved that the ArControl could record 200 Hz TTL signals from all six input channels simultaneously, and reaching up to 700 Hz when focusing on a single channel. The recording accuracy was ±1 ms (95% confidence, round effect; Figures 4C,D). The DAQ ability was powerful enough for behavioral research, since the maximal lick rate of mice was around 10–15 Hz.

## Behavioral experiments verification

Although we had technically verified the usability of the ArControl system, it was unclear if it was competent for practical behavioral paradigms. We focused on if the ArControl was adaptive for diverse behavioral tasks.

Two common behavioral tasks on mice, a Go/No-Go task (Gomez et al., 2007; Dolzani et al., 2013; Cui et al., 2017) and a two-choice procedure task (Tai et al., 2012; Stephenson-Jones et al., 2016), have been widely applied to assess discrimination and memory. Both tasks were modified slightly and used as verifications for the ArControl. All experimental data was acquired via the ArControl at Level 3, without any assistance from other DAQ devices. Detail hardware materials are shown in the supplements (Supplementary Figures S2A–C).

The experiment results of these two distinctive behavioral tasks were consistent with previous research. The data collected from experiments were adequate for offline analyses. Therefore, the ArControl was proven to be a reliable and a powerful platform to master multiple behavioral tasks.

### Go/No-Go behavioral task

**Figure 5:**
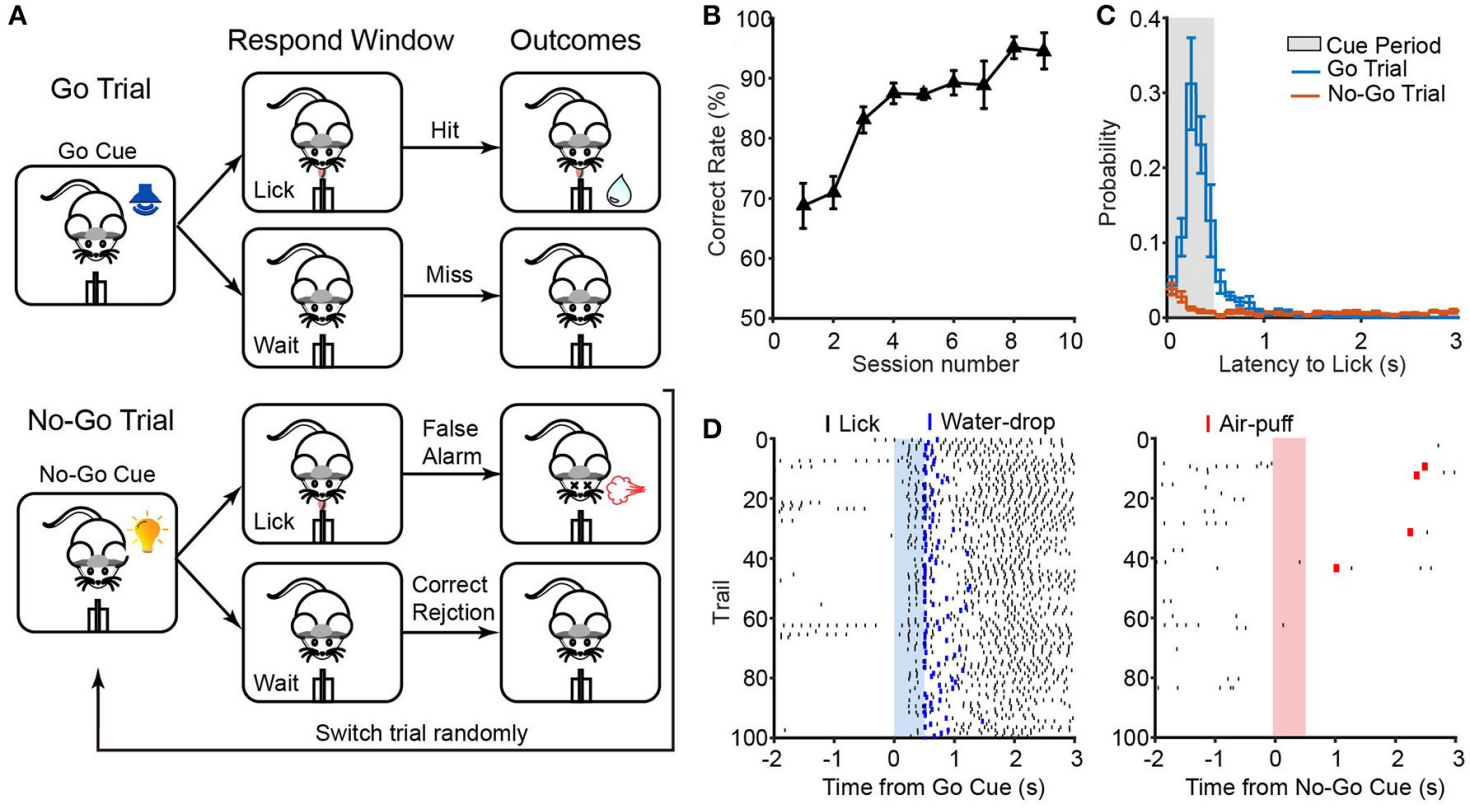
ArControl verification via the Go/No-Go task. **(A)** Sequence of events in a Go/No-Go task. **(B)** Learning curve across sessions (*n* = 5 mice). **(C)** Latency from the go/no-go cues onset to the first lick. **(D)** Licking behavior from a well-trained session aligned with go/no-go cues. Data are mean ± s.e.m.

Adult C57 head-fixed mice (*n* = 5) were trained with a Go/No-Go task. In this task, mice's bodies were restricted with a head bar and a body tube (Guo et al., 2014). They were required to discriminate a go cue (tone) and a no-go cue (light). They would consequently get a reward (water-drop) or a punishment (air-puff) once they responded (lick) to the go cue (tone) or the no-go cue (light) during a timed response window (Figure 5A).

Some typical parameters were calculated to assess the Go/No-Go model. The correct rate was a common index for evaluating the learning ability of mice (see Materials and Methods in Supplementary Material). After initial shaping, mice raised the correct rate from the upper chance level (70%) to the ceiling level (>90%) (Figure 5B). This learning process was similar to previous research (Liu et al., 2014). The response latency was another parameter that defined the time from cue onset to the first lick. The measurements of mice response showed that the well-trained mice were impulsive after the go cue and were patient after the no-go cue (Figures 5C,D). Furthermore, the recorded licking events were verified with gold standard (NI DAQ, sample rate = 1,000 Hz), which showed that the ArControl has no type I/II errors and possessed −0.6 ± 0.6 ms reliability (±*sd*; 4 sessions, 1,838 lickings).

### Probabilistic switching behavioral task

**Figure 6:**
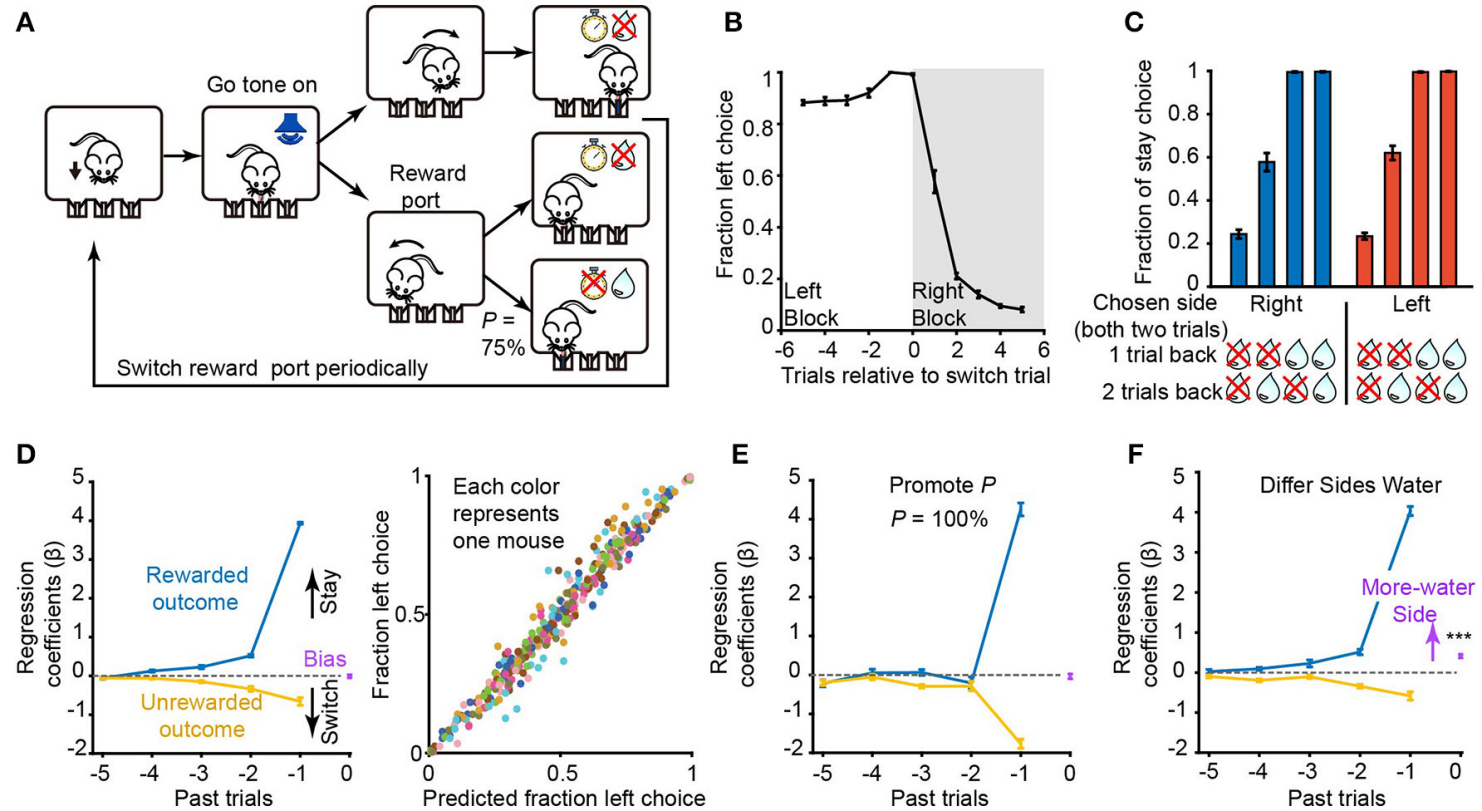
ArControl verification through the probabilistic switching task. **(A)** Sequence of events in a probabilistic switching task. **(B)** Fraction of choices for the left port (*n* = 8 mice) for trials before and after a switch of the rewarded port (at trial 0). **(C)** Fraction of stay choices for reward histories in which two consecutive choices were made to one port. **(D)** Left subplot: contribution of intrinsic bias (purple), rewarded (blue), and unrewarded (yellow) outcomes in the previous five trials on choices in current trial derived from the logistic regression. Right subplot: the actual fraction of choices for the left port plotted against the predicted fraction by the regression model (*n* = 8 mice, 43,000 trials totally). Data from each subject are grouped into 50 bins and represented by different colors. **(E)** Similar to **(D)**, but promotes the probability to 100 % (*n* = 8 mice, 16,000 trials totally). **(F)** Differ the reward size between ports (*n* = 4 mice, more water at left; *n* = 4 mice, at right; 30,000 trials totally). Data are mean ± s.e.m. Bias = 0.41 ± 0.04 mean ± s.e.m, Student's *t*-test, ^***^p < 0.001.

The spatial two-alternative forced-choice probabilistic switching task (2AFPC), a variant of the two-choice procedure task, requires participants to make a selection decision that relied on recent trial history. This was more challenging than the prior Go/No-Go task, since the ArControl should be able to handle more procedures and constraints. Free-moving adult C57 mice (*n* = 8) were trained in this task. The design of task followed the previous literature (Tai et al., 2012) with slight modification. The animals were required to initiate a trial by licking the central port, and sequentially move to a left or a right port in order to obtain a reward (Figure 6A). Only one port was rewarded by 75% at a time. In 25% of trials, neither port was rewarded. If no reward was delivered, animals would be punished by a time out. The rewarded port was periodically switched from time to time. The length of each block was randomized between 7 and 14 rewarded trials, and the switch only took place after a rewarded trial. Additionally, in order to prevent the mice from becoming demotivated when rewards were successively missing, the max consecution of reward-missing were limited to 2 trials consecutive.

After the initial training, mice took on average, 1.75 ± 0.07 (right to left) and 1.70 ± 0.07 (left to right) trials (±s.e.m., *n* = 8) to switch their behavior following the reversals between blocks (Figure 6B). The mice presented a win-stay/lose-shift strategy, based on the reward history in the previous trials, when making their next choice (Figure 6C). A regression model was fit to detail the mice's strategy, which indicated how the previous choice and the reward history determines the probability of the upcoming choice (Figure 6D).

The regression analysis revealed that the previous rewarded history had a positive effect on maintaining the current choice. The unrewarded history had the opposite, but weaker, effect (Figure 6D). Besides, the later trials had more weight than the earlier trials. These results corresponded to the previous research (Tai et al., 2012).

In addition, the role that some inherent parameters in the 2AFPC play in guiding mice behavior was investigated. First, the probability of reward delivered of correct trials was increased from 75 to 100%. The mice would definitely get a reward for every lose-shift case. This was consistent with our experimental results that the choices of mice were acutely relayed on the last trial (Figure 6E). Next, the reward size between the left and the right side was differentiated, resulting in a shift of bias point. There were no changes on the rewarded/unrewarded outcome curves (Figure 6F). It suggested that the reward size shifted the motivation between the left/right sides, not the discrimination of the mice.

## Discussion

The construction of behavioral platform is expensive, and becomes a barrier for groups with limited funds. Labs are forced to make a tough choice between buying expensive commercial platforms, and making a huge effort in adapting free systems. To address this problem, we developed a useful hardware and software combined platform, called the ArControl. We were the first researchers to combine the State Notation principle and the Arduino platform into a behavioral system. The ArControl is not merely a cheap and powerful alternative to commercial behavioral platforms. Meanwhile, the ArControl is superior to the commercial systems in that it was a genuine real-time system. Benefitted from this character, the ArControl was free from the computer load and achieved high temporal resolution (<0.1 ms, at Level 1; <1 ms, at Level 3). This temporal resolution was sufficient for behavioral research, since animal behavior is macro-scale against milliseconds.

There were still limitations for the ArControl. (1) Since the UNO is in the low end among the Arduino family, the maximum memory size of a behavioral task was 20 States. We are planning to make adaptation for higher end boards that would enhance the performance of ArControl. (2) Since the input and the output ports were ruled as TTL signals, the ArControl could only receive and generate simple signals. If users require a complicated output pattern, a better choice would be to design an extra signal generator, which could be connected to the ArControl through TTL communications. (3) The light DAQ module, which was a plug-in of the ArControl Level 3, was limited in that it could not record >200 Hz input signals. Otherwise, users should use a professional DAQ system and then perform hardware synchronization through a ready interface of ArControl. (4) Although adding code segments in the State was powerful and flexible, these segments did take away time from the DAQ module. A basic knowledge of the Arduino programming was still recommended when handling the complex schedules (e.g., the 2AFPC task). (5) The ArControl was incompetent for profound behavioral tasks, which required complicated interactions with computers or required visual signal processes. In this case, other toolboxes (Rose et al., 2008) were recommended.

We tested the performance of the ArControl from a technical assessment, as well as performing a head-fixed and a free-moving mice behavioral experiment. The ArControl was proven to be an economical, comprehensive, and reliable solution, especially for scientists who wish to construct behavioral platforms. Additionally, the ArControl had potential for accurately combining behavioral management and neuron operation. For example, the laser pattern in optogenetic is regularly customized among 5–30 Hz frequency and 5–10 ms duration. It was feasible that the ArControl could accurately control and/or record these laser events in a behavioral animal. Besides, the ArControl was a powerful developmental environment for the FSM. It could be used as a central logical manager for automation control of the time sequence of devices, such as streamline control, events detection, and instruments synchronization.

## Ethics statement

All procedures involving animals were approved by the Hubei Provincial Animal Care and Use Committee and the experimental guidelines of the Animal Experimentation Ethics Committee of Huazhong University of Science and Technology, China.

## Author contributions

HL: planned and organized the project; XC: designed the hardware and software; XC: performed the experiments; XC and HL: conceptualized the manuscript; XC: wrote the manuscript.

### Conflict of interest statement

The authors declare that the research was conducted in the absence of any commercial or financial relationships that could be construed as a potential conflict of interest.
