# Supplementary figures and images for: ArControl: An Arduino-Based Comprehensive Behavioral Platform with Real-Time Performance
Source: Front Behav Neurosci. 2017 Dec 11;11:244. doi: 10.3389/fnbeh.2017.00244 (PMC5732142; doi:10.3389/fnbeh.2017.00244)

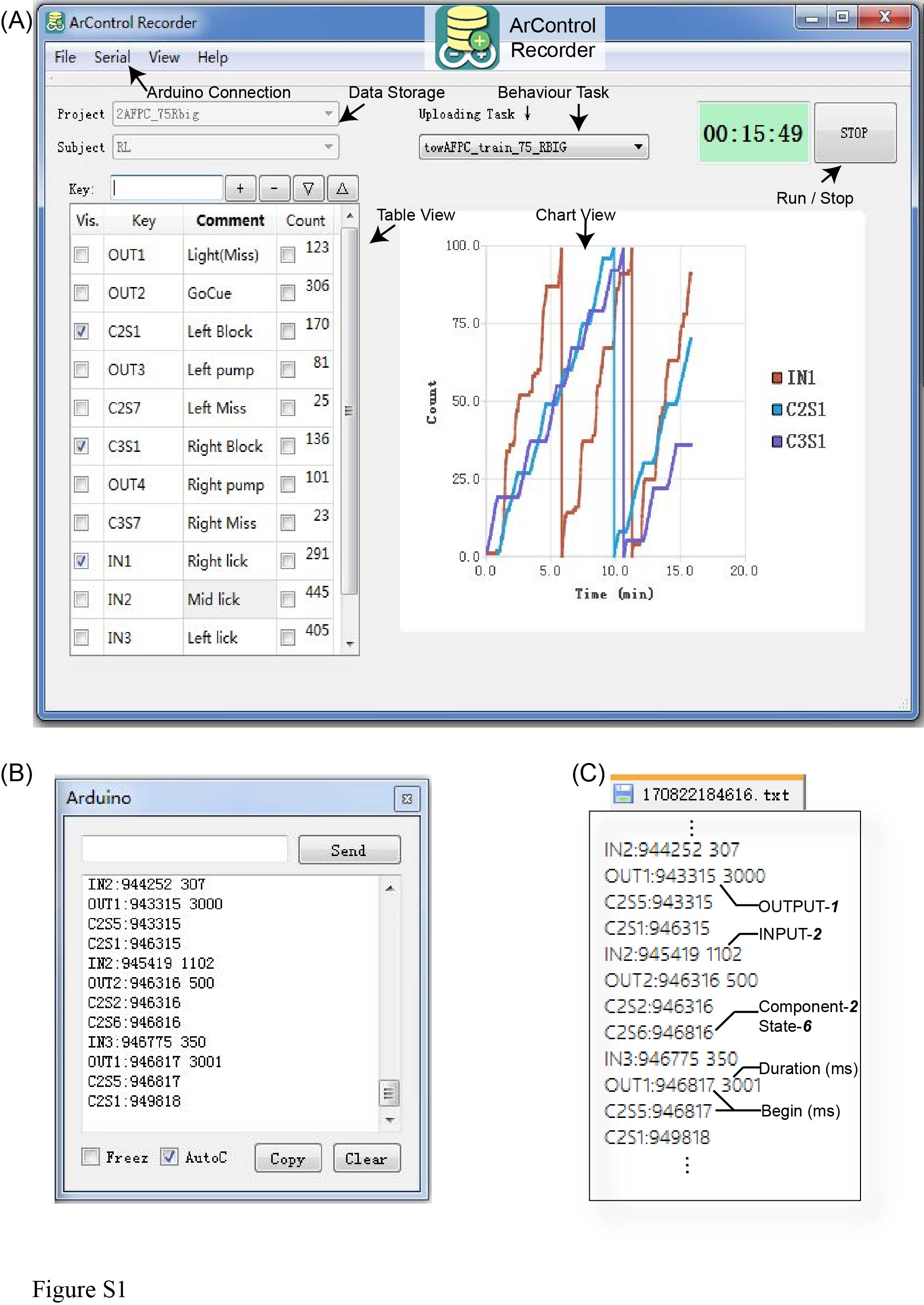

Supplement: Supplementary file 1 [file Image1.JPEG]

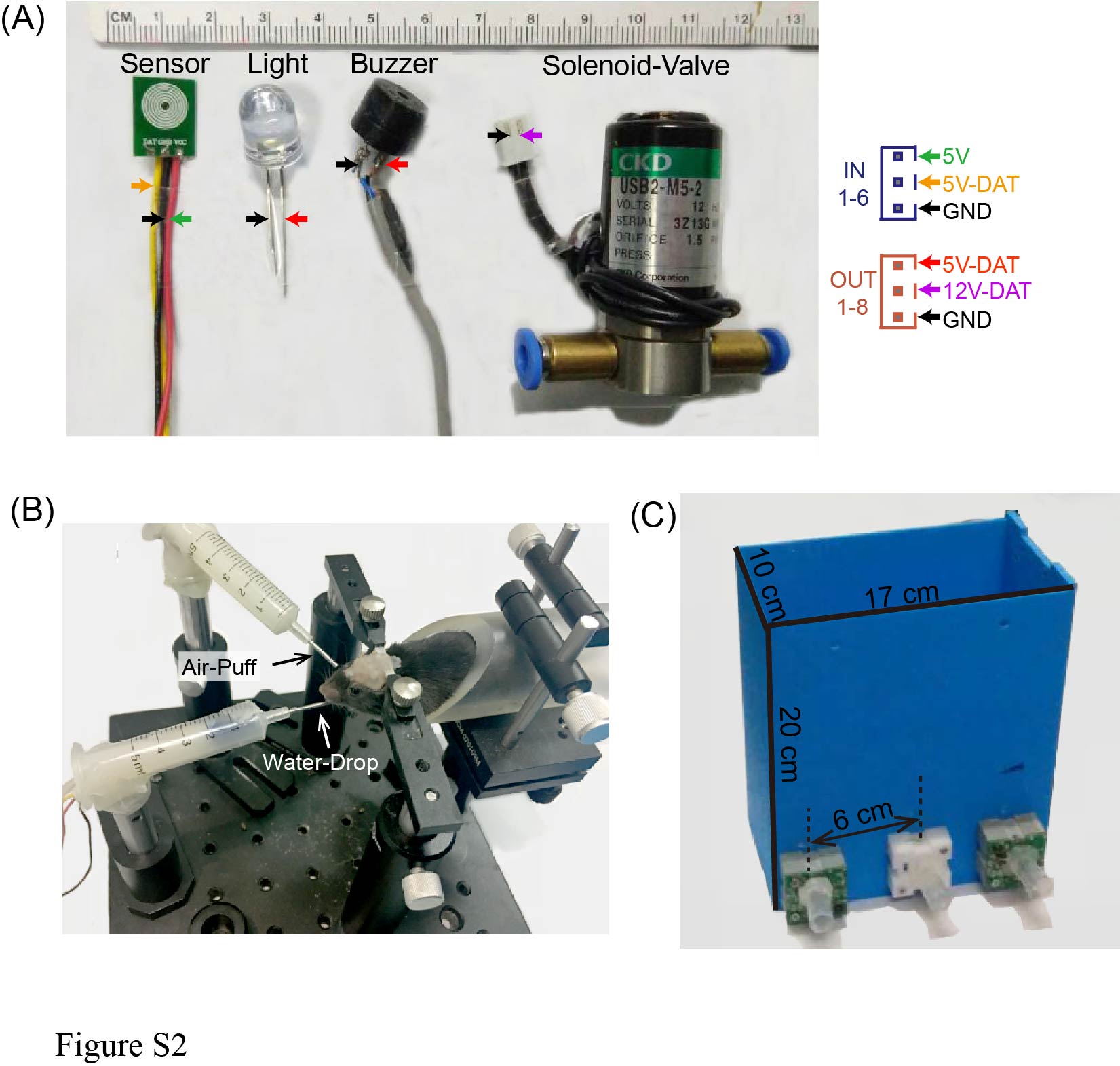

Supplement: Supplementary file 2 [file Image2.JPEG]
