# Supplementary material for: ArControl: An Arduino-Based Comprehensive Behavioral Platform with Real-Time Performance
Source: Front Behav Neurosci. 2017 Dec 11;11:244. doi: 10.3389/fnbeh.2017.00244 (PMC5732142; doi:10.3389/fnbeh.2017.00244)
Supplement: Supplementary file 3 [file Image3.PDF]

Supplementary Material

ArControl: An Arduino-based comprehensive behavioral platform with real-time performance

Chen Xinfeng1, Li Haohong*

*** Correspondence:** Li Haohong: hxli@hust.edu.cn

1 Materials and methods

1.1 Animals

Male C57BL/6 genetic background mice were used for the current study (4-12 weeks of age). Mice were group-housed under a 12-h light-dark cycle (7:00 to 19:00 light) and breed in the animal facilities at Wuhan National Laboratory for Optoelectronics. All procedures involving animals were approved by the Hubei Provincial Animal Care and Use Committee and the experimental guidelines of the Animal Experimentation Ethics Committee of Huazhong University of Science and Technology, China.

1.2 Go/No-Go behavioral training procedures

In the Go/No-Go discrimination learning task, head-fixed mice were placed in a sound attenuated chamber and trained to lick for acquiring water reward after go cue tone, while withholding response to a no-go cue light associated with punishment. Before training, mice were deprived water for 2 days reached 80%-85% of initial body weight. The task included shaping phase and training phase. Mice were trained for 2 sessions per day. Every session consists of 200-250 trials. In shaping phase, mice were trained for spontaneously licking water form a water tube at least 2 min (1-2 sessions). Then, water (5 μl) was delivered only mice licked the water tube in 2 seconds response window after go cue (tone, 0.5 s duration).

Typically in 1 to 2 sessions, mice could learn to lick for at least consecutive 20 trials paired with tone. Then the training phase was executed. Mice were trained to perform equal numbers of go/no-go trials in a random order. Air puff, water delivery, go cue, no-go cue, and licking events were triggered through serial ports and recorded by the ArControl. In go trials, water reward (5 μl) was delivered through a spout placed close to the mice mouth if mice lick in 2 s response windows after 0.5 s go cue tone. In no-go trials, licking resulted in a mild air puff (2 s) to the eye during the 2 s response window after no-go cue 0.5 s light. Inter-trial interval was random (4-6 s). The performance of each training session was defined by:

Correct Rate = (number of hit trials + number of correct rejection trials) / total number of trials

1.3 Probabilistic switch behavioral training procedures

In the spatial two-alternative forced-choice probabilistic switching task (2AFPC), free-moving mice were placed in an enclosed box. The location of a water reward was periodically switching at random

intervals. The initiation port was located in the middle of a wall, two choice ports were located 67.5 mm to the left and right of the initiation port (center to center; **Figure 6A**). A capacity-sensor/water-tube pair was placed on every side of the port to report the licks of mice and deliver water reward.

Every session consists of 200 rewarded trials. Mice initiated each trial by entering the center port or wait for 25 seconds, triggering Go tones instructing animals that the water was potentially available. Mice then chose a left or right peripheral port for water reinforcement (**Figure 6A**). The correct port was reward with a chance, and the incorrect and the reward-missed cases were penalized as 2 seconds break time with light instructing. Only after the set amount of rewards (7-14 for each block) was obtained and the last outcome was a reward (3 μL), the rewarded side could be switched to the opposite port. To prevent mice from getting demotivated when rewards are successively missed, the max consecution of reward-missing were numerically limited to 2 trials. Hence, the actual chance of reward from correct trials was a bit higher than the presupposition.

In the first stage (**Figure 6B-C**), the correct port delivered reward was presupposed to 75 % (actually 80±3 %). Data were rigidly extracted for each session, from the period when rewarded/total-trials was greater than 60 % at each port.

In the second stage (**Figure 6E**), the correct port delivered reward was presupposed to 100 % (actually 100 %). Data were rigidly extracted for each session, from the period when rewarded/total-trial was greater than 85 % at each port.

In the third stage (**Figure 6F**), the correct port delivered reward was presupposed to 75 % (actually 80±3 %), and the reward size differed for sides (2/4 μL). Data were rigidly extracted for each session, from the period when rewarded/total-trial was greater than 60%.

2 Supplementary Figures Legends

Supplementary Figure 1. Data collection from ArControl. (**A**) Main window of ArControl Recorder. (**B**) Serial monitor window of ArControl Recorder. (**C**) Matched segment extracted from data file.

Supplementary Figure 2. Additional hardware materials for behavioral box. (**A**) Input and output devices can be directly connected to driver hub. Solenoid-valve is used to give air-puff or water-drop. **(B)** Equipment for Go/No-Go task. (**C**) 3D printed box for two-choice procedure task.
